# A novel *FLCN* mutation in family members diagnosed with primary spontaneous pneumothorax

**DOI:** 10.1002/mgg3.1003

**Published:** 2019-10-18

**Authors:** Burcu Genc Yavuz, Esra Guzel Tanoglu, Seda Salman Yılmaz, Sahin Colak

**Affiliations:** ^1^ Department of Emergency Medicine University of Health Sciences Fatih Sultan Mehmet Training and Research Center Istanbul Turkey; ^2^ Department of Molecular Biology and Genetics University of Health Sciences Institute of Health Sciences Istanbul Turkey; ^3^ Department of Medical Genetics Cerrahpasa Medical School Istanbul University Istanbul Turkey; ^4^ Department of Emergency Medicine University of Health Sciences Haydarpasa Numune Training and Research Center Istanbul Turkey

**Keywords:** *FLCN*, mutation, pneumothorax

## Abstract

**Background:**

Primary spontaneous pneumothorax (PSP) is a disease characterized by the accumulation of air in the pleural space between the lung and thoracic wall. It is more common in young, tall, thin, and asthenic men. A family history was reported for approximately 11.5% of individuals admitted with PSP. The literature has reported cases diagnosed with familial PSP, who have no manifestations of Birt–Hogg–Dubé (BHD) syndrome but mutations in different exons of the *Folliculin* (*FLCN*) gene. The aim of this study is to present a Turkish family in which 13 members from three generations of the same family developed recurrent isolated spontaneous pneumothorax with a novel mutation in the *FLCN*.

**Methods:**

A male proband was diagnosed with spontaneous pneumothorax in the emergency department of the University of Health Sciences Haydarpasa Numune Training and Research Center, Istanbul, Turkey. His 12 relatives from three generations diagnosed with PSP, as revealed by his family history, were invited to the hospital to give blood samples for mutation analysis. The Sanger sequence data of *FLCN* were analyzed on the ENSEMBL website using SeqScape 3 and Codon Aligner software.

**Results:**

A novel heterozygous mutation c. 1273C>T (p.Gln425Ter) was detected in exon 11 of the *FLCN*, which caused PSP in the proband and his 12 relatives tested using Sanger sequencing.

**Conclusion:**

We found that a heterozygous mutation in exon 11 of *FLCN* c. 1273C>T (p.Gln425Ter), which was identified for the first time in our study, might cause isolated familial spontaneous pneumothorax.

## Graphical abstract

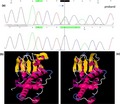

## 1 INTRODUCTION

Primary spontaneous pneumothorax (PSP) is a disease in which air is accumulated in the pleural space of individuals without underlying lung disease, not due to trauma and surgical intervention (Chiu & Garcia, 2006). It is more common in tall, thin, and asthenic men aged between 10 and 30. The incidence of PSP was 7.4–18/100,000 in men and 1.2–6/100,000 in women (Chiu & Garcia, 2006; Sahn & Heffner, 2000). PSP is considered to be caused by a rupture of subpleural blebs or bullae (Graham, Nolasco, Peterlin, & Garcia, 2005); however, the formation mechanism of blebs and bullae has not been clearly explained. A family history was reported for approximately 11.5% of individuals admitted with PSP (Graham et al., 2005). Coexisting monogenic disorders in PSP include alpha‐1 antitrypsin deficiency, Marfan syndrome, Ehlers‐Danlos syndrome, homocystinuria, and Birt–Hogg–Dubé (BHD) syndrome (Xing et al., 2017). The literature has reported cases with multiple PSP patients in the same pedigree who do not have the manifestations of these genetic disorders (Abolnik, Lossos, Zlotogora, & Brauer, 1991; Delaney, Gale, & Walker, 1974; Engdahl & Gershan, 1998; Gibson, 1977; Lenler‐Petersen, Grunnet, Jespersen, & Jaeger, 1990; Morrison, Lowry, & Nevin, 1998; Wilson & Aylsworth, 1979; Yamada, Takeda, Hayashi, & Shimizu, 2003).

Familial PSP was first defined by Faber in 1921 (Xing et al., 2017). Research has shown that the genetic inheritance of isolated PSP may be autosomal dominant, autosomal recessive, or X‐linked recessive (Fröhlich et al., 2008). The *folliculin *(*FLCN)* gene, a tumor suppressor gene localized in the short arm of the chromosome (17p11.2), was first identified in 2001 and reported to be associated with PSP, fibrofolliculoma, pulmonary cyst, and Birt–Hogg–Dubé Syndrome‐associated kidney cysts (Graham et al., 2005). Mutations in different exons of *FLCN* have recently been reported in patients who had no clinical manifestations of α1‐antitrypsin deficiency, connective tissue disorders, or Birt–Hogg–Dubé Syndrome but were only diagnosed with PSP (Fröhlich et al., 2008).

In the present study, we identified a novel and previously not reported mutation in *FLCN* in 13 individuals who had no clinical manifestations of α1‐antitrypsin deficiency, connective tissue disorders (e.g., Marfan syndrome or Ehlers‐Danlos syndrome), or BHD syndrome.

## 2 METHODS

### 2.1 Blood sampling

**Figure 1:**
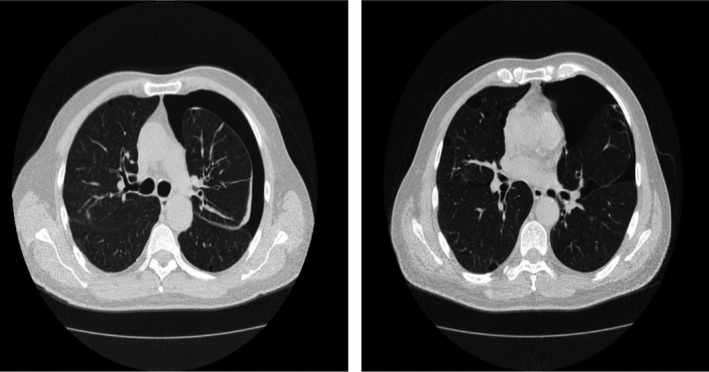
Chest high‐resolution computed tomography of the proband, who experienced recurrent episodes of PSP. Left‐sided PSP and bilateral and multiple bullae are seen as clear. PSP, primary spontaneous pneumothorax

A 60‐year‐old male proband of Turkish origin (II‐5) was diagnosed with spontaneous pneumothorax (Figure 1) in the emergency department of the University of Health Sciences Haydarpasa Numune Training and Research Center, Istanbul. His 12 relatives from three generations diagnosed with PSP, as revealed by his family history, were invited to the hospital. We evaluated when they had a pneumothorax attack, whether it was due to trauma or iatrogenic cause, and which therapies were used as well as the results of thoracic computed tomography. Blood samples were collected from affected individuals for *FLCN* mutation analysis.

### 2.2 Mutation analysis

Peripheral blood samples were collected into EDTA anticoagulant tubes and stored at +4°C until DNA isolation was performed. Genomic DNA was extracted from the blood samples of the proband case and family members in accordance with the instructions of the manufacturer. DNA isolation of the samples was performed using an automatic instrument kit (Qiagen, EZ1 Advanced XL). NanoDrop ND‐2000c (Thermo Fisher, Inc.) was used to quantify and assess the purity and concentration of isolated DNA samples. The DNA samples were stored at −20°C until the polymerase chain reaction (PCR) stage. The final amount of each PCR mixture was set to be 25 µl for the amplification of the *FLCN 4‐14* exons. DreamTaq PCR Master Mix (2X) (Thermo Scientific) was used in the PCR and the primer pair shown in Table 1 was used for *FLCN* (van Steensel et al., 2007). 100 μmol primers were diluted to 10 μmol and 0.5 µl was used per reaction. The following touchdown PCR conditions were used: 94°C for 5 minutes followed by 16 cycles of 94°C for 30 s, 68°C for 30 s, 72°C for 1 minute; then 25 cycles of 94°C for 30 s, 55°C for 30 s, 72°C for 1 minute, and 72°C for 10 minutes. The PCR products were electrophoresed on a 2% agarose gel followed by PCR purification by ExoSAP (GML). The DNA sequencing reaction was performed using the BigDye Version 1.1 (Life Technologies) and the sequencing was performed using the 3500 Genetic Analyzer (Applied Biosystems). The sequence data were analyzed on the ENSEMBL website using SeqScape™ Software v3.0 and Codon Aligner Software 3.0 with the ENST00000285071 transcript. We also used Jmol Software 13.0 program to show whether any conformational change in protein structure of the *FLCN* ("Jmol: an open‐source Java viewer for chemical structures in D.").

**Table 1:** List of primer sequences for polymerase chain reaction amplification

Primer name	Primer sequences 5’→3’
*FLCN4F*	5'‐AGG TGC TCC CTG TGC TCC AG‐3'
*FLCN4R*	5'‐CCG TCC ACT GCT CTC AGG TC‐3'
*FLCN5F*	5'‐CCG AGC TCA GAT TTG CAT AAA CC‐3'
*FLCN5R*	5'‐CCT GCC TCC CTG TGC AAT G‐3'
*FLCN6F*	5'‐TGA TTT GTG CCA GCT GAC TCT G‐3'
*FLCN6R*	5'‐CCA GGC CTC AAC CTC AGC AC‐3'
*FLCN7F*	5'‐CCT GGA GTT GGC TGT GAA CG‐3'
*FLCN7R*	5'‐TCC CAA ATC CAT GGA CAA GC‐3'
*FLCN8F*	5'‐GTT GTG CCC TGC TGG TGT TC‐3'
*FLCN8R*	5'‐TTC CCT CCC TCA GCG ATT CC‐3'
*FLCN9F*	5'‐GGC CGC AGC CAG GAA TCT/AC3'
*FLCN9R*	5'‐GTG GAG GGT CCA GAG GCA AG‐3'
*FLCN10F*	5'‐CAC CCG CCT CCC TGA GAA G‐3'
*FLCN10R*	5'‐CCA GTG GAG ACC GTG TGG TG‐3'
*FLCN11F*	5'‐GGT TCC ACT TTG GGC CTG AG‐3'
*FLCN11R*	5'‐AGG AGG CGT GTG GGG TTT G‐3'
*FLCN12F*	5'‐CTA GCG CAG GGG AGG TGA GG‐3'
*FLCN13R*	5'‐ACG GCC CAG CTC CTC TTT TG‐3'
*FLCN14F*	5'‐CCG TGT CAC CCC TGG TTG G‐3'
*FLCN14R*	5'‐TGC TGG GAC ACA GCT CCT TC‐3'

## 3 RESULTS

**Figure 2:**
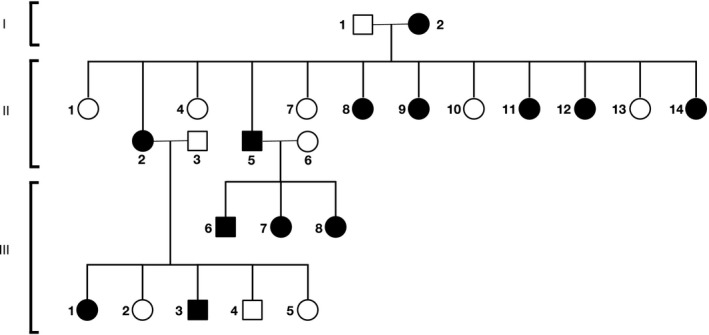
Pedigree of the Turkish family with primary spontaneous pneumothorax. Circles are females, squares are males, affected individuals are highlighted in black. Roman numbers indicate generations and Arabic numbers indicate individuals. The index patient is II‐5

In our study, PSP was detected in 13 cases, including the proband. We found that 11 of the family members underwent invasive treatment and two family members underwent conservative treatment due to PSP. Figure 2 shows the pedigree of the family members included in the study. The skin examination showed that none of the patients had fibrofolliculoma skin lesions. The ultrasound imaging of the urinary system showed that they had no renal mass. Additionally, none of the patients had clinical manifestations of BHD syndrome or a connective tissue disease such as Marfan syndrome and Ehlers‐Danlos syndrome. In the patients, α1‐antitrypsin deficiency was excluded. Among 13 patients included in the study, 77% (*n* = 10) were female and 23% (*n* = 3) were male. The patients had a total of 32 pneumothorax attacks. The mean age at the first pneumothorax attack was 40.23 years and the mean BMI was 25.55 kg/m^2^. The median number of episodes was 2.46. All 13 patients had bilateral and multiple air cysts (bullae and blebs) on thorax CT images. All patients had normal respiratory function tests. None of the patients had interstitial lung diseases or diffuse parenchymal lung diseases such as bronchiectasis and tuberculosis.

The proband's mother (I‐2) underwent chest tube drainage when she was 68 years old due to left PSP. She had no recurrence so far. In the second generation, six of 11 sisters (II‐2, 8, 9, 11, 12, and 14) had a history of PSP. In the third generation, three children (III‐6, 7, and 8) of the proband and two children (III‐1 and 3) of one of the six sisters (II‐2) had a history of PSP. There was no consanguineous marriage in the first generation and the parents of the third generation had no ties of kinship. Table 2 displays the clinical data of 13 family members with a history of PSP. None of the family members had a known history of hereditary disease or cancer.

**Table 2:** Clinical data of affected with familial spontaneous pneumothorax

	I−2	II−2	II−5	II−8	II−9	II−11	II−12	II−14	III−1	III−3	III−6	III−7	III−8
Age (years)	87	68	60	55	55	53	52	47	45	42	36	35	31
Sex	F	F	M	F	F	F	F	F	F	M	M	F	F
Age at first attack of PSP	70	68	46	31	50	48	34	38	36	27	22	29	24
No. of PSP attack	1	1	5	3	2	1	1	1	3	3	6	2	3
PSP location	L	R	B	B	B	L	L	R	B	B	B	B	B
BMI (kg/m^2^)	27.7	26	26.8	28.6	29.7	22.3	28.7	26.3	20	28.4	23.1	22.6	22
Pulmonary bullae/blebs on CT scan	B&M	B&M	B&M	B&M	B&M	B&M	B&M	B&M	B&M	B&M	B&M	B&M	B&M
Treatment	TD	OBS	TD + Op	TD + Op	TD + Op	TD + Op	OBS	TD	TD + Op	TD + Op	TD + Op	TD	TD + Op

Note

Abbreviations: B&M, bilateral and multiple; B, bilateral; BMI, body mass index; CT, computed tomography; F, female; L, left; M, male; OBS, observation; Op, surgery operation; PSP, primary spontaneous pneumothorax; R, right; TD, tube drainage.

**Figure 3:**
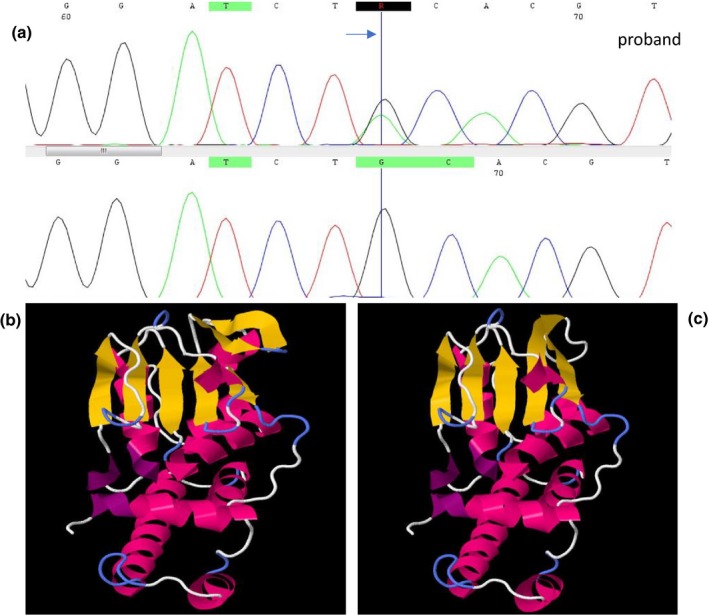
Chromatographs of sequence analysis of *FLCN* in proband and parents (a). A schematic diagram showing the wild‐type (b) and altered (c) protein structure in the residue where the mutation resides. *FLCN*, folliculin

A novel heterozygous mutation c. 1273C>T (p.Gln425Ter) was detected in exon 11 of *FLCN* of the proband tested using Sanger sequencing. The novel mutation c. 1273C>T was also present in the family members (Figure 3). The deleterious annotation of genetic variants (DANN) analysis performed using VarSome Clinical revealed a DANN score of 0.9977 for the mutation we identified. This mutation results in a substitution of glutamine with terminal mutation which is a highly conserved residue among several species. Our analysis showed that conformational change in the mutated residue using Jmol 13.0 Software to compare the wild‐type and the mutant protein structure of *FLCN* (Figure 3).

## 4 DISCUSSION

In this study, we identified, for the first time, a nonsense mutation, which has not been previously reported in individuals diagnosed with PSP in the literature, in the *FLCN* of 13 members from three generations of the same family with a history isolated PSP.

To date, studies have reported the presence of more than 100 germline mutations in 14 exons of *FLCN* (Lim et al., 2010). These mutations in the *FLCN* have been identified in patients with BHD syndrome and patients with isolated familial PSP.

It has been reported that 50% of mutations in the *FLCN* are frameshift mutations resulting in a stop codon, while splice site, nonsense, missense, and deletion mutations are less prevalent (20%, 14.3%, 8.6%, and 4.3%, respectively) (Kim, Yoo, Kang, Cho, & Lee, 2012).

In this study, a heterozygous nonsense mutation was detected in exon 11 of *FLCN* of 13 members from three generations of the same family diagnosed with PSP. The c. 1273C>t mutation has not been reported as a variation in databases such as the Human Gene Mutation Database, 1000 Genomes, and Exome Variant Server. We believe that the single‐nucleotide mutation located in the c. 1273C>T (p.Gln425Ter) region of the *FLCN* result in a stop codon, thereby leading to protein truncation and losses of function. The DANN score ranges from 0 to 1 and a DANN score of 1 represents the highest possibility for pathogenicity. The DANN score of the identified mutation was found to be 0.9977.

Some mutations have been reported in different exons of the *FLCN* in cases of isolated familial spontaneous pneumothorax (Table 3). An inframe deletion mutation has been detected in exon 6 of *FLCN* in a Korean family with a history of recurrent PSP (Kim et al., 2012). A heterozygous C deletion (c.+ 1285) mutation in exon 11 has been found in the genetic analysis of an Indian family with a history of PSP (Ray, Paul, Chattopadhyay, Kundu, & Roy, 2015). A heterozygous mutation in exon 7 of *FLCN* was detected in a Swiss pedigree with a history of PSP (Fröhlich et al., 2008).

**Table 3:** Reported mutations and their localizations in *FLCN* of familial spontaneous pneumothorax patients

Gene	Mutations	Exon	Mutation type	Family phenotype	References
*FLCN*	c.1429C>T	12	Nonsense mutation	PSP	(Graham et al., 2005)
*FLCN*	c.943G>T	9	Nonsense mutation	PSP	(Graham et al., 2005)
*FLCN*	c.733delTCGG	4	Frameshift	PSP	Painter, Tapanainen, Somer, Tukiainen, & Aittomäki, 2005)
*FLCN*	c. 510C>G	6	Nonsense mutation	PSP	(Zhu, Shen, Zhu, & Tian, 2017)
*FLCN*	c.779G>A	7	Nonsense mutation	PSP	(Fröhlich et al., 2008)
*FLCN*	c.394G>A	5	Missense	PSP	(Fröhlich et al., 2008)
*FLCN*	c.924_926del	6	Inframe deletion	PSP	(Ren et al., 2008)
*FLCN*	c.1611_1631del	10	Frameshift	PSP	Ren et al., 2008)
*FLCN*	c.1740C>T	11	Missense	PSP	(Ren et al., 2008)
*FLCN*	c.1733insC	11	Frameshift	PSP	(Ren et al., 2008)
*FLCN*	c.1285delC	11	Frameshift	PSP	(Ray et al., 2015)
*FLCN*	nt1988 del GATG	13	Frameshift	PSP	(Gunji et al., 2007)
*FLCN*	nt1733 ins C	11	Frameshift	PSP	(Gunji et al., 2007)
*FLCN*	nt857 del C	6	Frameshift	PSP	(Gunji et al., 2007)
*FLCN*	nt852 ‐1 del gtccctccag	intron 5	Inframe deletion	PSP	(Gunji et al., 2007)
*FLCN*	nt1795 ins CCACCCT	12	Frameshift	PSP	(Gunji et al., 2007)
*FLCN*	c.1537 del‐C	10	Deletion mutation	PSP	(Sundaram, Tasker, & Morrell, 2009)
***FLCN***	**c. 1273C>T**	**11**	**Nonsense mutation**	**PSP**	*Our mutation

Abbreviation: *FLCN*, folliculin.

Mutations in exons 9 and 12 of *FLCN* have been reported to be associated with a higher number of cysts, larger cysts, and a higher incidence of pneumothorax (Toro et al., 2007). The frequency of renal neoplasm has been reported to be significantly lower in cytosine deletion mutations in exon 11 (Xing et al., 2017). Considering the previous studies, no definite distinction has been found between the type of *FLCN* mutation, BHD syndrome, and familial spontaneous pneumothorax. Therefore, it has been suggested that familial PSP cases should be evaluated in terms of BHD syndrome and the presence of fibrofolliculoma and renal mass should be excluded (Menko et al., 2009). In our study, the diagnosis of BHD syndrome was excluded because none of the patients had a history of fibrofolliculoma, renal cancer, or a different malignancy. *FLCN* mutations may also lead to different malignancies because it functions as a tumor suppressor gene. Possible genetic mutations that may cause isolated familial spontaneous pneumothorax are today still investigated. There is a need for further research on the subject.

In conclusion, we found that a heterozygous mutation in exon 11 of *FLCN* c. 1273C>T (p.Gln425Ter), which was identified for the first time in our study, might cause isolated familial spontaneous pneumothorax. The data suggest that autosomal dominant inheritance may be responsible for this transition. The presence of this mutation also confirms the familial inheritance of PSP in the proband.

We think that the causes underlying the molecular mechanism of familial spontaneous pneumothorax can be discovered through the examination of the connection between mutations in different exons of the *FLCN* and familial spontaneous pneumothorax.

## CONFLICT Of INTEREST

None declared.

## AUTHOR CONTRIBUTIONS

BGY and SC had the original idea for the study, BGY and EGT designed it. BGY sought institutional approval, compiled, checked and analyzed the data, managed the study database, computed the results, and wrote the initial draft of the manuscript including the text, tables, and figures. SSY and BGY together worked on the statistical analyses. BGY, EGT, SSY, and SC all critically reviewed the study design, contributed to the collection and analysis of the data and the interpretation of mutations and results, and commented on and approved the final manuscript. BGY submitted the study and is responsible as the guarantor for the overall content.

## PATIENT CONSENT FOR PUBLICATION

The informed consent form was obtained from each family member involved in the study.

## ETHICS APPROVAL

University of Health Sciences Haydarpasa Numune Training and Research Center (KAE).
